# Changes of Colonic Bacterial Composition in Parkinson’s Disease and Other Neurodegenerative Diseases

**DOI:** 10.3390/nu10060708

**Published:** 2018-06-01

**Authors:** Sara Gerhardt, M. Hasan Mohajeri

**Affiliations:** Departement of human medicine, University of Zurich, Winterthurerstrasse 190, 8057 Zürich, Switzerland; sara.gerhardt@uzh.ch

**Keywords:** Parkinson’s disease, gut microbiome, neurodegenerative diseases, microbiota–gut–brain axis

## Abstract

In recent years evidence has emerged that neurodegenerative diseases (NDs) are strongly associated with the microbiome composition in the gut. Parkinson’s disease (PD) is the most intensively studied neurodegenerative disease in this context. In this review, we performed a systematic evaluation of the published literature comparing changes in colonic microbiome in PD to the ones observed in other NDs including Alzheimer’s disease (AD), multiple system atrophy (MSA), multiple sclerosis (MS), neuromyelitis optica (NMO) and amyotrophic lateral sclerosis (ALS). To enhance the comparability of different studies, only human case-control studies were included. Several studies showed an increase of *Lactobacillus*, *Bifidobacterium*, Verrucomicrobiaceae and *Akkermansia* in PD. A decrease of *Faecalibacterium* spp., *Coprococcus* spp., *Blautia* spp., *Prevotella* spp. and Prevotellaceae was observed in PD. On a low taxonomic resolution, like the phylum level, the changes are not disease-specific and are inconsistent. However, on a higher taxonomic resolution like genus or species level, a minor overlap was observed between PD and MSA, both alpha synucleinopathies. We show that standardization of sample collection and analysis is necessary for ensuring the reproducibility and comparability of data. We also provide evidence that assessing the microbiota composition at high taxonomic resolution reveals changes in relative abundance that may be specific to or characteristic of one disease or disease group, and might evolve discriminative power. The interactions between bacterial species and strains and the co-abundances must be investigated before assumptions about the effects of specific bacteria on the host can be made with certainty.

## 1. Introduction

The causes of the heterogenic group of neurodegenerative diseases (NDs) are still unknown but several contributing factors including genetic and lifestyle factors and age-related aberration of health are likely to play a role. The pathology in PD was found to start in more caudal parts of the central nervous system (CNS) or even in the enteric nervous system, bringing the gut and its interaction with CNS into the spotlight [1,2,3]. While some researchers question whether PD begins in the gut, a potential role of the gut microbiome in PD pathology is undisputed [4]. In the intestinal tracts of PD patients, a pro-inflammatory microbiota composition was discovered, which might lead to increased gut permeability. This high permeability of the intestinal mucosa is known as leaky gut, since bacterial products and inflammatory mediators could pass through the gut’s mucosa and invade the blood [5].

PD is a neurodegenerative disease with the hallmark of cardinal progressive motor symptoms such as tremor, muscular rigidity, postural instability and bradykinesia, mostly additionally accompanied by nonmotor symptoms. Characteristic are furthermore the alpha synuclein-containing aggregations, named Lewy bodies, that can be found in the central, autonomous and enteric nervous system [6].

Indeed, the microbiota greatly modulates the function and homeostasis of the gut and the human health beyond the gut. There are up to 100 times more microbial genes, named the microbiome, than human genes in one individual [7]. The largest part of humans’ microbiota is gathered in the gut and the community compromises of more than 10 trillion cells and up to 1000 different microbial species per individual [8]. This complex community is responsible for a myriad of different metabolic, immunologic and homeostatic functions [9].

Which special bacterial strain is responsible for which function in the colon is difficult to determine as evolution favoured functional redundancy to create a diverse and stable ecosystem in the gut [10]. The gut bacteria species are able to co-exist by resource partitioning and niche differentiation. Most gut bacteria metabolise by fermentation, since the majority of resources are derived from host-ingested carbohydrates [10]. It will be of importance to study the functions of pioneer species and community formation to understand the effects of microbiome manipulation in modulating human disorders [10]. One of the most beneficial functions of gut microbes is the synthesis of various vitamins. Some Bifidobacterial species, belonging to the phylum of Actinobacteria, can produce folate, while lactobacilli appear to be unable to synthesize folate de novo. *Bacillus subtilis*, a species belonging to the Firmicutes, and *Escherichia coli*, a Proteobacterial species, are both able to synthesise riboflavin. *Propionibacterium freudenreichii*, a species belonging to the phylum Actinobacteria, is used in the commercial production of vitamin B12. Also, *Lactobacillus reuteri*, another species from the Firmicutes phylum, is supposed to be capable of vitamin B12 synthesis [11]. Presenting the recent research topic of immunological functions and interactions of gut microbiome with the host are challenging fields of research that are the subject of dedicated reviews [12,13,14].

The different taxa interact, rely on and crowd out each other, leading to distinct compositions in various stages of human health and disease [15,16] and age [17,18]. In animal studies different potential mechanisms of neurodegenerative disease were discovered, where the gut microbiome played a key role in brain physiology and function. The gut–brain axis is a bidirectional communication pathway comprising direct neural, endocrine and immunological mechanisms [19]. The gut–brain axis may be implicated in alpha synuclein-mediated pathology, which is supposed to spread from the gut to the brain [20]. Also, in humans, there is some evidence supporting the role of the gut–brain axis in PD pathophysiology, for example that truncal vagotomy reduces the risk for PD [21]. Moreover, a healthy intestinal barrier function seems crucial for maintaining neurological health [22]. In animal models of NDs a distinct and discriminative microbial composition was found, e.g., for ALS [23,24] and AD [25]. Consequently, some attempts were made to assess the microbial composition in human patients suffering from neurodegenerative diseases [26,27]. For this purpose, different methodical approaches were used such as 16S rRNA or 16S cDNA sequencing, metagenomic shotgun sequencing and different microarray assessments.

This review aims to summarize changes in the microbiota composition assessed by these genetic investigations in human patients with NDs. This is, to our knowledge, unique in the broad coverage of findings of microbiome in patients of different NDs. Since the largest part of gut microbiota is comprised of bacteria and their role in NDs is supported by the most scientific evidence, while the term also includes fungi and viruses, this review concentrates on the compositional changes of the bacterial taxa. We show that analysis of colonic bacterial composition at the highest possible resolution may be predictive for PD and discriminate PD from other neurodegenerative diseases [19,28,29,30,31,32]. Methodical standardization procedures ensure the comparability of studies.

## 2. Materials and Methods

The key question of this review was whether PD patients present specific taxonomic changes in gut microbiota discriminating them from other NDs and from healthy controls. This is a systematic review of human case control studies, summarizing the findings of original studies published in the last five years (01.01.2013–31.12.2017), during which the most research on this topic has been performed.

PubMed databank and online books were searched due to combining the MESH terms of the following NDs (a JPND, EU Joint Programme—Neurodegenerative Disease Research: Parkinson’s disease, Alzheimer’s disease, prion disease, motor neurone diseases, Huntington’s disease, spinocerebellar ataxia, spinal muscular atrophy and additionally multiple system atrophy, multiple sclerosis, neuromyelitis optica, amyotrophic lateral sclerosis) combined with following terms: “microbiome”, “microbiota”, “bacteria”, “bacterial”, “composition”, intestine, “intestinal”, “gut”.

We concentrated on bacterial taxa and excluded results regarding fungi, archaea and viruses.

Articles that fulfilled the following criteria were included in the comparison:

- subjects in the case groups had a diagnosis for the disease given by an expert.
- human case control studies
- at least one part of the study analysed the gut microbiome in a cross-sectional manner compared to healthy controls
- faeces collection to generate a sample probe
- microbiota analysis by amplification sequencing methods or hybridisation on microarrays: Phylochip G3, YIFscan
- published in peer-reviewed journals
- paper available in English language

**Figure 1:**
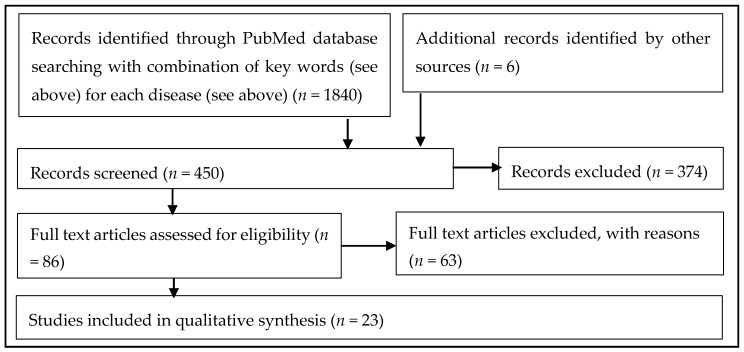
Methodical approach of systematic review due to PRISMA criteria (PRISMA criteria [33]).

Animal studies were excluded because our review did not aim at proving a possible mechanistic explanation of neurodegeneration (see Figure 1). Our aim was rather identifying the exact taxa that play a role in NDs, because such information may be used as potential biomarkers and thus be clinically relevant for early disease diagnosis. Thus, the potentially confounding influences of different host species are ruled out by comparing only human data. The focus of our review is PD, but the results of all mentioned NDs can be seen in the additional Table A1 in the Appendix. To our knowledge no other review to date provides such a comprehensive overview of the observed relative microbiome changes in abundance in PD as well as in other NTs such as Alzheimer’s disease (AD), multiple system atrophy (NSA), multiple sclerosis (MS), neuromyelitis optica (NMO) and amyotrophic lateral sclerosis (ALS).

## 3. Results

Ten case control studies enrolling PD patients were included in the analysis. Five studies used the Illumina Miseq sequencing methods [34,35,36,37,38], while two studies used Illumina Hiseq sequencing method [39,40]. One study used the Roche 454 GS FLX Titanium sequencing method [41]. Two included studies used other taxonomic identification and quantification methods: Yakult Intestinal Flora-SCAN (YIF-Scan) [42] or 96-well block of the ABI PRISM 7900HT Sequence Detection System [43]. The last two methods only assess targeted microbial taxa and are used to reproduce previously reported findings, whereas the methods of the other eight studies assess the whole microbiome and may detect surprising unknown species. Since BMI, age and gender are expected to influence the gut microbiome, a table with information about the main clinical characteristics of the study participants from the 10 included Parkinson’s studies is provided (Table 1).

In the majority of publications, where information on Alpha diversity/richness was given, no significant difference between healthy controls and PD cases was found. However, in all reports where information on overall beta-diversity was presented, it differed significantly between healthy controls and PD (Table 2).

### 3.1. Taxonomic Changes

Taking the 10 publications together, changed relative bacterial abundances between HC and PD were observed on all taxonomic levels. A table is provided for each phylum, which showed changes between HC and PD in at least one study. Only significant changes (*p* < 0.05), which underwent a statistical analysis and correction by the authors of the original study, were included in the tables. The tables only show changes on phylum, family, genus, species or operational taxonomic unit (OTU) levels, always in the highest taxonomic resolution, which the authors of the study provided. The few additional changes on class or order level will be specifically described in the discussion. Significant changes in relative abundances were observed only within five phylae: Firmicutes, Actinobacteria, Bacteroides, Verrucomicrobia and Proteobacteria.

#### 3.1.1. Firmicutes

The most changes in relative abundance of colonic microbial taxa were observed within the Firmicutes phylum. Single studies found changes in unclassified Firmicutes and in various families and genera (Table 3). A significant increase of genus *Lactobacillus* [34,35,42], decrease of genus *Faecalibacterium* or species *Faecalibacterium prausnitzi* or OTUs that belong in this category [34,35,37,43], decrease of genus *Coprococcus* [38] or species *Coprococcus eutactus* [34] or OTU [35] belonging in this category and decrease of the genus *Blautia* [37,38] or species *Blautia glucerasea* [34] or OTU belonging in this category [35] were reported and confirmed in PD.

#### 3.1.2. Actinobacteria

In all studies in which changes in relative abundance of or within the phylum Actinobacteria were reported, PD patients reproducibly showed an increase in relative abundance compared to HC [34,35,37,43] (Table 4). The increase of the genus *Bifidobacterium* was reproduced in three studies [34,35,43].

#### 3.1.3. Bacteroidetes

The published data for the phylum Bacteroidetes was highly variable between the different reports (Table 5). The potential trend of decreased Bacteroidetes on phylum level [37,43] and decreased genus *Bacteroides* [34] or species *Bacteroides fragilis* [42] was observed in the opposite direction by one publication [38]. Three studies found a significant reduction of Prevotellaceae family [32,43] or the genus *Prevotella* [34,39]. One study found a similar but not significant decrease of Prevotellaceae [43]. Hill-Burns et al. (2017) did not confirm these findings, but observed an increase in one OTU that belongs to the genus *Prevotella* [35].

#### 3.1.4. Verrucomicrobia

Like as within the phylum of Actinobacteria, also in Verrucomicrobia all reported changes were increases of certain microbial taxa [35,38,39,40,41] (Table 6). Within the phylum Verrucomicrobia there was a high overlap of the results from the different PD studies. The relative abundance of Verrucomicrobiaceae family [35,38,40,41] and *Akkermansia* genus [35,38,39,40] were increased in four studies.

#### 3.1.5. Proteobacteria

Single studies found changes in relative abundance within the Proteobacteria, Alpha-proteobacteria- and Gammy-Proteobacteria phylae. None of these findings were reproduced by other studies, apart from the increase in the Enterobacteriaceae family in two studies [37,43] (Table 7).

## 4. Discussion

Our data show that the reproducibility of data is very low at a higher taxonomic rank, the phylum level. The inconsistency of the data could indicate that the changes of phyla are facultative but not obligatory for NDs. Moreover, it implies that it is sufficient for an effect on the host that lower taxonomic levels are altered without achieving an alteration at phylum level. Profound changes in one family could be compensated for by oppositional changes in other families of that phylum. In conclusion, comparisons on phylum level are too rough, and higher taxonomic resolution is needed to discover similarities of the microbiota composition relating to the diseases.

A negative correlation between PD disease duration and Firmicutes abundance was reported [38]. Additionally, an inverse correlation was found between the COMT inhibitor type of PD medication with Entacapone and Firmicutes abundance [43]. These findings show that there is more to PD than a lower Firmicutes phylum abundance, since otherwise Entacapone would not display its beneficial effects. No other study apart from Keshavarzian et al. (2015) found a significant change in abundance of Firmicute phylum in PD [38]. This implicates the importance of controlling disease medication and disease duration of the subjects during the study since both alter the gut microbiota composition. For the aim of proving causality, a reduction of confounding may be achieved by enrolling a study population consisting of PD patients at an early stage or/prodromal stage and no medication.

Since the Firmicutes:Bacteroidetes ratio is found to change with age [44], an association with neurodegeneration could be considered. However, no study reported any significant differences in Firmicutes:Bacteroidetes ratio in PD. A minority of studies in other NDs, namely two ALS studies [45,46] and one MSA study [47], discovered an aberrant Firmicutes:Bacteroides ratio, indicating no predominant role for the NDs. Moreover, several other factors influence the Firmicutes:Bacteroides ratio. The characteristics of the recruited cohort are crucial. In the elderly but also in infants, the ratio is extremely low [44]. It was suggested that the Firmicutes:Bacteroidetes ratio is also associated with obesity [48,49,50,51], but a causative key role for this ratio for obesity was questioned by other groups [52]. Different types of diet change the Firmicutes to Bacteroides ratio [53], and it was reported that freezing faecal samples before extracting the DNA significantly increases the Firmicutes:Bacteroides ratio [54]. Other methodical choices, like choosing between the different bead beating cell lysis instruments before metagenome analysis, influence this ratio up to threefold in favour of Bacteroides [55]. Taken together, these data indicate that the shift is not specific for neurodegenerative diseases and might sometimes be a consequence of technical and lifestyle factors.

Several alterations in the Firmicutes phylum were reported in PD and other NDs. Commensal *Clostridium* species, like *Clostridium saccharolyticum* and *Clostridium leptum*, showed a significant and descriptive decrease in PD [42]. This reduction is in agreement with the observed reduction of the genus *Clostridium* in AD [56] and MS [57]. By contrast, an increase of the harmful species *Clostridium perfringens* was shown in one study in NMO patients [58]. This is a very interesting finding, since NMO is a demyelinating disease that is associated with antibodies against the water channel protein aquaporin-4 (AQP4) expressed on astrocytes and T helper 17 cells [59]. These antibodies are reported to exhibit a cross-reactivity to a homologous peptide sequence within the adenosine triphosphate-binding cassette (ABC) transporter permease of *Clostridium perfringens* [59,60]. Also, a theoretical involvement of the epsilon toxin of *Clostridium perfringens* in inducing neurodegeneration was proposed in MS due to its ability to cross the blood–brain barrier, damaging oligodendrocytes and due to its observed contribution to enterotoxaemia in ruminants [61,62,63]. However, no increased *Clostridium perfringens* was found in the five MS studies [57,64,65,66,67]. Nonetheless, epsilon toxin might play a role in NMO since it can damage optic nerves in vivo [68]. This and the possibility to vaccinate against epsilon toxin, at least in rodents, point to an importance of the observed increase of *Clostridium perfringens* in NMO [69].

*Lactobacillus* was increased in three of 10 PD studies, in contrast to AD [56], MSA [47,70], ALS [45,46,71] and NMO [58], where no change was observed. However, this increase of *Lactobacillus* in PD patients could be caused by the frequent constipation of PD patients, since *Lactobacillus* is also known to be increased in constipation-type IBS and decreased in diarrhoea-type IBS [72] and the co-variable constipation was methodically and statistically treated differently in the PD studies. The decrease of both *Lactobacillus* and *Lactobacillus rogosae* that was reported in MS patients [57,66] supports the above hypothesis, since several medications against MS lead to diarrhoea [73,74]. The possibility that a probiotic intervention or preventive treatment with beneficial *Lactobacillus* strains could have an ameliorating effect on MS deserves to be investigated.

Increased Lactobacillaceae abundance and decreased *Prevotella* has been linked to reduced ghrelin concentration and altered ghrelin secretion has been reported in one PD study [43]. Ghrelin is a gut hormone that may contribute to the maintenance and protection of normal nigrostriatal dopamine function [74]. Future studies should investigate if *Lactobacillus* has a constipation-related consecutive role, or rather a causative role. Such studies could, moreover, define which *Lactobacillus* species are beneficial and which may be detrimental. This differentiation is essential since there are probiotic treatment proposals for PD that include *Lactobacillus* strains and, on the other hand, the potentially harmful link with reduced ghrelin secretion has not been sufficiently investigated.

Two PD studies showed an increase of Ruminococcaceae family [31,35], which might also discriminate PD from other NDs, since in other NDs either no change or even a decrease was observed, namely in AD [56], MSA [47], MS [75] and ALS [46]. The increased Ruminococcaceae on a family level might play a role in PD patients; however, which subordinate taxa contribute to the disease cannot be unequivocally stated yet.

*Faecalibacterium*, partly specified as *Faecalibacterium prausnitzi* or as an out, showed a significant decrease in four PD studies [34,35,37,43], while this was not observed in any other ND apart from MS [57,65]. In ALS an increase was noted in one study [76]. However, *Faecalibacterium* showed a negative correlation to Entacapone taxa [43] and a positive correlation to vitamin D supplementation in MS patients [65], indicating that also other factors might contribute to the observed change of *Faecalibacterium* abundance. Thus, the specific *Faecalibacterium* change in PD might be provoked by extrinsic factors including medication or nutrition. Nevertheless, *Faecalibacterium prausnitzi* is advocated as a potential future probiotic due to some strain-dependent anti-inflammatory features, like butyrate production [77]. However, the hypothesized beneficial effects on intestinal barrier integrity resulting in anti-inflammatory benefits could not be confirmed by one study in an in vitro model of the large intestine [78]. Since the *Faecalibacterium prausnitzi* species include a high diversity of strains, a strain-dependent classification and investigation would improve insight into the beneficial and harmful effects on the host [79].

Lachnospiraceae family showed a depletion not only in PD [35], but also in MSA [47], MS [75] and ALS [45]. Even if this reduction on family level is not specific to PD, it is still interesting, because Lachnospiraceae include many putative anti-inflammatory and thus potentially protective genera. In addition, Lachnospiraceae were negatively correlated to disease duration in PD patients [38]. Thus, this reduction might just be a consequence of common disease-related changes. The key members of Lachnospiraceae showed a confirmed decrease in PD (Table 3). Interestingly *Blautia* and *Dorea* are also decreased in MSA [47], while AD [56], NMO [58] and MS [66] showed an increase of *Blautia* and MS [66] and ALS [45] a decrease of *Dorea*. On a family level, the decrease of Lachnospiraceae is correlated with PD disease duration and the abundance of Lachnospiraceae might be, like the incidence of PD, sex-dependent [36]. Concluding that the decrease of *Blautia* and *Dorea* in case-control-studies is limited to alpha-synucleinopathies like PD and MSA, it is tempting to investigate, if these genera additionally correlate with longitudinal data of PD and MSA patients and if the changes predate the neurodegenerative progress and symptoms.

Another family, Christensenellaceae, showed an increase on family and species level, e.g., *Christensenella minuta*, during the course of PD [34,35], a change that was also observed by one study on genus level of *Christensenella* in MS patients [75]. Thus, these changes might not be specific but still characteristic for some PD patients. Same applies to the increase of *Oscillospira* in PD [34,38]. Petrov et al., moreover, explained recently that Christensenellaceae is a heritable taxon that is co-abundant with *Oscillospira*. These taxa are, like PD, significantly associated with lower body weight [80], which might indicate a higher vulnerability for PD, or more severe PD stages, when these families were inherited.

Two different species of *Eubacterium* were observed to be decreased in PD, MS and a case-control-study of a cognitive impaired elderly without an explicit AD diagnosis [39,57,81]. In the cognitively impaired elderly, a negative correlation between *E. rectale* and proinflammatory cytokines IL-1b, NLRP3 and CXCL, in addition to a positive correlation between these cytokines and *Escherichia/Shigella*, was observed [81]. This might imply an anti-inflammatory role for several Eubacteria species in the development of neurodegenerative diseases. Regarding changes in the metabolome of colonic microbiota, *Eubacterium* contributed the most to the decrease in genes for D-glucuronate degrading enzymes and two more active tryptophan metabolism pathways observed in PD patients [39]. Glucuronidase enzymes mediate the regeneration of molecules important for host health, but also toxins and carcinogens [82]. Moreover, they activate endogenous glucuronides of hormones and neurotransmitters [82]. l-Tryptophan is the precursor of serotonin and is shown to be decreased in PD patient brains [39] and its metabolites display regulatory immune function with beneficial and harmful potential . Finally, some non-significant trends, between Unified Parkinson’s disease rating scale III (UPDRS III) and abundances of e.g., *Eubacterium rectale, Eubacterium hallii* and *Eubacterium eligens* were reported [39]. This is mentionable since *E. rectale* might penetrate the mucus layer due to its flagella. Interactions between *E. rectale*, the mucus layer and the intestinal mucosa are therefore feasible.

The members of the phylum of cyanobacteria, which belong to the blue-green algae, produce a series of neurotoxins, from which beta-N-Methylamino-l-alanine (BMAA) is the most common. However, the production of neurotoxins by their candidate phylum sibling Melainabacteria is not yet well investigated [83]. A link between cyanobacterial derived neurotoxins and neurodegenerative diseases was shown already more than 10 years ago by Cox et al., who examined the Chamorro people of Guam, who exhibit a 50–100 times higher incidence of ALS than anywhere else [84]. BMAA is linked with intraneuronal protein misfolding, which is an important pathological hallmark of many NDs. High levels of neurotoxin BMAA have been associated with AD [85]. Post-mortem brain specimens of ALS, AD and Huntington’s disease (HD) patients showed increased BMAA concentrations [86]. Moreover, BMAA targets *N*-Methyl-d-aspartate (NMDA) receptors and the neurotransmitter glutamate, which are believed to be disturbed in AD. Also, other cyanobacterial derived neurotoxins could play a role in neurodegeneration in individuals with more permeable intestinal epithelial barrier of the GI tract, like the leaky gut observed in elderly people [87]. However, none of the examined studies detected an altered relative abundance in Cyanobacteria in PD. Also, in other neurodegenerative diseases like AD [56], MSA [47,70], MS [57,64,65,66,76], ALS [45,46,71] and NMO [58], no change in this phylum was reported. Only Heintz-Buschart et al. reported a decrease of an unclassified OTU171 with high resemblance to the Melainabacterium MelB1,57 in PD. However, they could not asses any of the known cyano-neurotoxins [40]. This and the fact that this Melainabacterium was decreased in PD speak in favour of a protective outcompeting role, possibly by replacing other harmful cyanobacteria. Products of other bacteria, belong to the phylum of red algae, such as a phycoerythrin, were shown to have an ameliorating effect on AD [88,89]. It can be summarized that Cyanobacteria can produce neurotoxins that lead to neurodegeneration, but the abundance of Cyanobacteria is only increased in special, sometimes isolated populations. The role of the newly detected phylum of Melainabacteria is not yet clear, but so far no harmful association has been found and a protective role can even be postulated.

Only a few changes were reported in the phylum of Actinobacteria, but these were consistent regarding several taxonomic resolutions (Table 4). PD patients showed an increase on phylum level [37], which was reflected in an increase in Bifidobacteriaceae (family) [35] and *Bifidobacterium* (genus) [34,35,43], while no changes were observed in any other examined NDs. In contrast, AD patients showed a decrease on a phylum level, reflected by a decrease in Bifidobacteriaceae (family) and *Bifidobacterium* (genus) [56]. Despite the findings of an increased Bifidobacteriaceae in PD, a two-year follow-up study showed that a decrease in *Bifidobacterium* later in disease may be able to predict whether PD stage is going to deteriorate or not, even correlating with the Unified Parkinson’s disease rating scale I (UPDRS I) score [90]. This implicates that the increase in Bifidobacteriaceae in PD is not detrimental to patients, but rather may represent a mechanism of beneficial action against neurodegenerative aggravation. Thus, it may be postulated that a probiotic intervention with *Bifidobacterium* could prevent progression of PD to severer stages.

Within the phylum of Bacteroidetes, *Prevotella* is a well-known and often discussed genus. However, changes in PD patients were not consistent throughout the PD studies (Table 5). In one study, which failed to find any significant difference in relative *Prevotella* abundance between PD subjects and controls, *Prevotella* still was the most reduced bacterium and differed by 3.2-fold [42]. In another study without a statistically significant change in *Prevotella*, an association between the family Prevotellaceae and the clinical score of PD severity, the UPDRS-III score, was reported [41]. Moreover, trends showing a decrease in *Prevotella* were obtained by one study when looking at the microbiome of colonic mucosa rather than faeces [38]. Negative results concerning significant changes in *Prevotella* could be a consequence of methodological differences and other criteria for control subjects [38]. The fact that Prevotellaceae was decreased in idiopathic rapid eye movement behavioural sleep disorder, iRBD, which predates PD in a majority of cases, supports the hypothesis that this family and its genus *Prevotella* might only be changed in the early stages of PD. It is also suggestive that a decrease of *Prevotella* contributes to the onset of PD and therefore could be used as a biomarker for PD diagnosis [40]. However, unambiguous interpretation of changes of *Prevotella* is difficult, since their abundance also depends on social factors, as observed in monkeys [91], and subgenus differences in *Prevotella* and *Bacteroides* have been related to different dietary patterns [92].

Additionally, the change in abundance of *Prevotella* depends on the species type (Table 5). *Prevotella copri* and *P. clara* were decreased in PD [34,39,57]. This reduction is in agreement with reduced abundances in MSA [70], MS [64] and NMO [58]. In contrast, the species *Prevotella melaninogenica* and one OTU of *Prevotella* showed an increase in PD [39] and NMO [58]. *Prevotella* exhibited one of the highest effect sizes of change in the study of NMO [58]. The fact that certain *Prevotella* species (especially *Prevotella copri*) are indeed decreased in all NDs, apart from AD (see Table A1 in the Appendix), suggests a possible protective role against neurodegenerative processes in the brain and deserves future investigation.

In the Verrucomicrobia phylum, four studies found that *Akkermansia* genus or species were increased in PD patients [35,38,39,40]. Moreover, both high colonic Akkermansia abundance and PD were shown to be negatively correlated to BMI [93]. *Akkermansia muciniphila* exerts beneficial effect on the intestinal mucosal layer and improves the barrier function of the gut epithelium [39]. Thus, in conditions of low *Akkermansia* abundance, the maintenance of a healthy gut barrier may not be possible anymore, and pathogenic factors of other bacteria, like Lipopolysaccharide (LPS), could consequently harm the host. On the other hand, *Akkermansia* uses mucus as an energy source and degrades the mucus layer [94]. This could lead to increased exposure of microbial antigens to immune cells and thus could have inflammatory potential. The role of the pro- and anti-inflammatory properties is not yet clear but maintaining a steady state of *Akkermansia* may be a pre-requisite for normal functioning of the gut homeostasis. *Akkermansia* together with *Eubacterium*, *Capnocytophaga*, *Phascolarctobacterium* and OTUs no further classified than to Firmicutes, achieved a high prediction score for PD [39].

No changes within the phylum of Proteobacteria, apart from the family Enterobacteriaceae [37,43], were reported in PD patients. Therefore, it can be concluded that the relative abundance of these taxa is only changed in a minority of patients. Because of the missing confirmation of these alterations, the clinical relevance of these findings can be questioned. *Escherichia coli*, a member of the Enterobacteriaceae family, showed translocation into the colonic mucosa in PD patients [95]. The complexity of this interrelationship is shown by the increased abundance of Enterobacteriaceae in PD patients with the postural instability and gait difficulty (PIGD) phenotype compared to the tremor-dominant subtypes (TD) [41]. The more progressive and severe pathology of non-TD-phenotypes (including PIGD) supports the idea of a different unknown pathological mechanisms underlying this subgroup of PD [41]. The observed difference in Enterobacteriaceae abundance between the subgroups of PD disease might equally occur due to other variable phenotype features in this study: PIGD subjects tended to be older and to have higher nonmotor symptoms scale (NMSS) total scores than TD subjects [41]. These differential abundant taxa between PD phenotypes was challenged by another study [43] as these researchers only found an overall increase in PD [37,43]. However, in this study Unger et al. used a slightly different classification and they interpreted their results based on a small number of the TD patients in their sample. In agreement with the findings in PD, a significant increase of Enterobacteriaceae and some other taxa of Proteobacteria phylum was also observed in NMO. The mentioned change in Enterobacteriaceae was not only significant but they were also four times more abundant than in healthy controls [58]. A protective role of Enterobacteriaceae was proposed by Hasegawa et al., demonstrating that Enterobacteriaceae (and *C. perfringens, B. fragilis*, and *Pseudomonas*) produce hydrogen water (1000 mL/day) and were associated with improved total UPDRS scores in PD patients in a double-blind randomized controls study [96]. In addition, they were shown to be protective against PD pathology in rats [97]. These researchers performed a study to assess whether the total amount of breath hydrogen in PD patients was lower than in healthy controls and confirmed this hypothesis [42]. The results regarding the potential protective or aggravating role of Enterobacteriaceae are contradictory and require further investigation.

A high number of microbiota composition changes found in human case-control studies of ND concern unclassified bacteria. Approximately 40% of the gut microbiome cannot be captured by reference genome-based methods [98]. It is hypothesized that during ND unclassified bacteria gain more importance. Heintz-Buschart et al. discovered 10 depleted unclassified OTUs in PD patients that exhibited together very low fractional abundance [40], but could still manipulate the host’s health via an unknown unique mechanism without a need for high abundance. Interestingly, the genome of OTU 469 encoded an endoglucanase with a synuclein-like domain and thus may lead to an immune system that is tolerant to alpha-synuclein-like structures [40]. This could have a potential implication for the induction of PD and MSA pathology. The depletion of this bacterium leads to a lack of host’s immune system exposure to the bacterial alpha-synuclein-like structures, which may result in immunologically induced alpha-synuclein aggregation. This hypothesis is in line with the reports that alpha-synuclein aggregation starts in the enteric nervous system and not in the brain [1,3,99]. All unclassified OTUs of this study, where the genome could be recovered, showed common features such as fermentative lifestyle, motility and the ability to synthesize vitamins. The great number of observed changes in unclassified Bacteria or single OTUs must be handled carefully since the method of quantification of the most included studies was 16S rRNA amplification and high-throughput sequencing, which is known to prone to sequencing errors and chimera creation, leading to an artificial high diversity and low reproducibilityLastly, no studies have analysed the gut microbiome of iRBD patients yet (apart from Heintz-Buschart et al.), but this disease showed a high conversion rate to alpha-synuclein disorders, especially PD. More investigations of microbiome in prodromal stages of diseases with follow-up evaluation are necessary.

Only one human case control study analysed the colonic microbiome of AD patients [56]. Most AD patients were in the stage of mild or very mild AD according to clinical dementia rating (CDR) scores. Targeting the early stage of the AD may help to examine possible causal associations between microbiome compositional changes and disease onset. Limited overlap was found between changes of the microbiome in AD and changes identified in any of the 10 PD studies. Minor similarities were observed that included a decreased Firmicutes phylum and Clostridium genus in AD, whereas significantly decreased *Clostridium leptum* [42] or *Clostridium saccharolyticum* [39,42] was observed on PD. Moreover, AD showed an increased Bacteroidetes phylum, *Bacteroides* genus [38] and Alistipes genus, while increased *Alistipes shahii* [39] was evident in PD patients. As the above findings were only found in a minority of the cohorts, no great similarity between the microbiome of AD and PD can be determined so far.

## 5. Conclusions

Changes in the composition of colonic microbiota are found in PD and several other NDs [26,27]. Some evidence has emerged that assessing the microbiota composition at high taxonomic resolution can lead to specific alterations in relative abundance for one disease or disease group that might evolve discriminative power. For example, decreased *Blautia* and *Dorea* is limited to alpha synucleinopathies like PD [34,35,37,38] and MSA [47]. Inheriting increased Christensenellaceae and co-abundant increased *Oscillospira* might predispose for a higher vulnerability for PD or more severe PD stages [34,38], and *Prevotella* reduction occurs in early PD stages and might function as a biomarker for PD [40]. A definitive concluding rating of the importance of these changes for the development and diagnosis of NTs, however, is difficult to date. The reasons lie in methodological discrepancies in collecting and analysing the microbiome as well as varying sample and effect sizes. Moreover, other confounders such as genetics, medication, nutrition and lifestyle factors may result in insignificant or falsified results. Therefore, methodical standardization is necessary for ensuring the collection of comparable, reliable and reproducible data. To compare microbiota composition between studies and diseases, it is most straightforward to first assess the known microbiota via more reliable microarray methods and additionally make a functional analysis. In a next step, one may try to detect new species via 16S rRNA sequencing. Finally, the functional variability within one species and the change of features due to plasmid exchange deserves more attention. The interactions between bacterial species and strains and the co-abundances must be more deeply investigated before assumptions about the effects of specific bacteria on the host can be made.

## Figures and Tables

**Table 1:** Demographics and clinical data of Parkinson’s disease (PD) cases and human controls (HC).

Reference PD/HC	[42]	[43]	[34]	[35]	[36]	[37]	[38]	[39]	[40]	[41]
*n*	52/36	34/34	89/66	197/130	29/29	24/24	38/34	31/28	76/78	72/72
Gender %male	40.4/58.3	70.6/52.9	--	67.0/39.2	79.3/44.8	66.7/42.9	63.2/52.9	100	66/59	51.4/50.0
Mean Age	68.9/68.4	67.7/64.6	67/63	68.4/70.3	69.2/69.4	73.8/74.6	61.6/45.1	64.8/65.6	68.0/68.4	65.3/64.5
Mean BMI * Median BMI	20.2/22.6	--	26.7/26.1	26.4/28.3	--	23/24	26.0/27.6	--	28.5/26.6	26.3/26.2 *

This table shows the main demographic and clinical data of the participants in the 10 Parkinson’s studies. -- indicates that no information was found in the original publication; * Median BMI.

**Table 2:** Richness, alpha diversity, beta diversity.

Reference	[42]	[43]	[34]	[35]	[36]	[37]	[38]	[39]	[40]	[41]
Indexes/Method	YF	PR	Ill Miseq					Ill Hiseq		Ro
Faecal bacterial counts	>	n	n	n	n	n	n	n	n	n
Alpha diversity/Richness on at least one taxonomic level (Chao 1 index, *other indexes)	n	n	>	n	-	-	<*	-	n	-
Overall Beta diversity (weighted Unifrac, °other indexes)	n	n	sd	sd	sd °	sd °	n	°		sd

This table shows the significant differences in faecal bacterial counts, alpha diversity/richness and beta diversity between healthy controls (HC) and Parkinson’s disease (PD). > symbolizes a higher abundance in HC when compared to PD; < symbolizes a lower abundance in HC when compared to PD; - symbolises that no statistical significant difference was found between HC and PD; sd indicates a statistically significantly difference; n symbolizes, that no information was given. The methods used by the studies are Yakult Intestinal Flora-SCAN (YF), 96-well block of the ABI PRISM 7900HT Sequence Detection System (PR), Illumina Miseq sequencing (Ill Miseq), Illumina Hiseq sequencing (Ill Hiseq), Roche 454 GS FLX Titanium sequencing (Ro). * symbolzises that other alpha diversity indexes where used, like Shannon, Simpson and Richness (Margalef) [38]. ° symbolzises, that other beta diversity indexes where used, like unweighted Unifrac [36], Shannon [37] or univariate tests [39]

**Table 3:** Firmicutes.

Reference	[42]	[43]	[34]		[35]	[36]	[37]	[38]	[39]	[40]	[41]
Taxa/Method	YF	PR	Ill Miseq						Ill Hiseq		Ro
Unclassified Firmicutes (unclass.)									<		
Firmicutes unspecified (unspec.)								>			
Lactobacillaceae unspec.		>			<	<					<
Lactobacillus, m = mucosae, g = gassero, c = caseo, f = fermentum, re = reuteri, ru = ruminis,	<(+g, c, f, re, ru)		<(+m)		<						
Enterococcaceae unspec		>				<	<				
Enterococcus							<				
Ruminococcaceae unclassified					<						
Ruminococcaceae OTU 4439469					<(o1)						<
Ruminococcus, b = bromii, c = callidus			<(b)	>(c)			>				
Papillibacter c = cinnamivorans			<(c)								
Faecalibacterium, p = prausnitzi		>(p)	>		>(o2)		>				
Lachnospiraceae unclassified					>(o3)						
Lachnospiraceae unspec					>						
Roseburia					>(+o2)			>			
Coprococcus, e = eutactus			>(e)		>(o1)			>			
Blautia, g = glucerasea			>(g)		>(+o1)		>	>			
Dorea, l = longicatea			>(+l)								
Catabacteriaceae Catabacter, h = honkongenesis			<(+h)								
Clostridiaceae Anaerotruncus										<	
Clostridium, c = coccoides, s = saccharolyticum	>(c)								>(s)		
Eubacteriaceae, [candidatus stoquefichus massiliensis]			>								
Erysipeltrichoceae unspec.							<				
Eubacterium, b = biforme									>(b)		
Christensenellaceae unclass.					<						
unspec					<						
Christensenella, m = minuta			<(+m)								
Oscillospiraceae, oscillospira			<					<			
Streptococcaceae, unspec							<				
Streptococcus							<				
Acidaminococcaceae, Acidaminococcus							<				
Veillonellaceae unspec							<				
Veillonellaceae, Megamonas							<				
Veillonellaceae Megasphera							<				
[Tissierellaceae] unspec.					<						

This table shows the significant relative changes in the Firmicutes phyla between PD patients (PD) and healthy controls (HC), that were assessed by the 10 summarized Parkinson’s disease studies. > symbolizes a higher abundance in HC when compared to PD; < symbolizes a lower abundance in HC when compared to PD; An empty cell symbolizes, that no significant change in PD compared to HC was found with the chosen methodological approach. o1, 2, 3 symbolize, that one, two, or three OTUs of the taxa were significantly changed in PD compared to HC; + indicates that a change in PD compared to HC on the genus and on the species level was found. The methods used by the studies are Yakult Intestinal Flora-SCAN (YF), 96-well block of the ABI PRISM 7900HT Sequence Detection System (PR), Illumina Miseq sequencing (Ill Miseq), Illumina Hiseq sequencing (Ill Hiseq), Roche 454 GS FLX Titanium sequencing (Ro).

**Table 4:** Actinobacteria.

Reference	[42]	[43]	[34]	[35]	[36]	[37]	[38]	[39]	[40]	[41]
Taxa/Method	YF	PR	Ill Miseq					Ill Hiseq		Ro
Unspec						<				
Bifidobacteriaceae, Unspec.				<						
Bifidobacteriaceae, Bifidobacterium		<	<	<						
Coriobacteriaceae, Unspec						<				

This table shows the significant relative changes in Actinobacteria between PD patients (PD) and healthy controls (HC), that were assessed by the 10 summarized Parkinson’s disease studies. < symbolizes a lower abundance in HC when compared to PD; Empty cells symbolize, that no significant change in PD compared to HC was found with the chosen methodological approach.The methods used by the studies are Yakult Intestinal Flora-SCAN (YF), 96-well block of the ABI PRISM 7900HT Sequence Detection System (PR), Illumina Miseq sequencing (Ill Miseq), Illumina Hiseq sequencing (Ill Hiseq), Roche 454 GS FLX Titanium sequencing (Ro).

**Table 5:** Bacteroidetes.

Reference	[42]	[43]	[34]	[35]	[36]	[37]	[38]	[39]	[40]	[41]
Taxa/Method	YF	PR	Ill Miseq					Ill Hiseq		Ro
Unspec		>				>	<			
Bacteroidaceae, Bacteroides c = coprocola, d = dorei, f = frgailis, p = phlebeus, m = massiliensis	>(f)		>(+m, c, d, p)				<			
Prevotellaceae, Unspec										>
Prevotellaceae, Prevotella, co = copri			> (+co)	<(o1)				>(+co)		
Porphyromonadaceae, Parabacteroides				<						
Porphyromonadaceae, Barnesiella (in the original article classified as Barnesiellaceae)					<					
Rickenellaceae, Alistipes, s = shahii								<(s)		

This table shows the significant relative changes in Firmicute phyla between PD patients (PD) and healthy controls (HC), that were assessed by the 10 summarized Parkinson’s disease studies. > symbolizes a higher abundance in HC when compared to PD; < symbolizes a lower abundance in HC when compared to PD;empty cells symbolize, that no significant change in PD compared to HC was found with the chosen methodological approach . o1symbolizes, that one OTU of the taxa were significantly changed in PD compared to HC; + indicates that a change in PD compared to HC on the genus and on the species level was found. The methods used by the studies are Yakult Intestinal Flora-SCAN (YF), 96-well block of the ABI PRISM 7900HT Sequence Detection System (PR), Illumina Miseq sequencing (Ill Miseq), Illumina Hiseq sequencing (Ill Hiseq), Roche 454 GS FLX Titanium sequencing (Ro).

**Table 6:** Verrucomicrobia.

Reference	[42]	[43]	[34]	[35]	[36]	[37]	[38]	[39]	[40]	[41]
Taxa/Method	YF	PR	Ill Miseq					Ill Hiseq		Ro
Unspec									<	
Verrucomicrobiaceae, Unspec				<			<		<	<
Akkermansiaceae, Akkermansia, m = muciphila				<			<	< (+m)	<	

This table shows the significant relative changes in Firmicute phyla between PD patients (PD) and healthy controls (HC), that were assessed by the 10 summarized Parkinson’s disease studies. < symbolizes a lower abundance in HC when compared to PD; empty cells symbolize, that no significant change in PD compared to HC was found with the chosen methodological approach. + indicates that a change in PD compared to HC on the genus and on the species level was found. The methods used by the studies are Yakult Intestinal Flora-SCAN (YF), 96-well block of the ABI PRISM 7900HT Sequence Detection System (PR), Illumina Miseq sequencing (Ill Miseq), Illumina Hiseq sequencing (Ill Hiseq), Roche 454 GS FLX Titanium sequencing (Ro).

**Table 7:** Proteobacteria.

Reference	[42]	[43]	[34]	[35]	[36]	[37]	[38]	[39]	[40]	[41]
Taxa/Method	YF	PR	Ill Miseq					Ill Hiseq		Ro
Unclassified							<			
Alpha-proteobacteria, Bradyrhizobiaceae, unspec										<
OTU 469 (o1 = one OTU)									>	
Gamma-Proteobacteria, Enterobacteriaceae, Unspec		<				<				
Gamma-Proteobacteria, Enterobacteriaceae, Escherichia/Shigella						<				
Gamma-Proteobacteria, Enterobacteriaceae, Proteus						<				
Gamma-Proteobacteria, Moraxellaceae, Unspec						<				
Gamma-Proteobacteria, Moraxellaceae, Acinetobacter						<				
Gamma-Proteobacteria, Pasteurellaceae, unspec.				>						

This table shows the significant relative changes in the Firmicutes phyla between PD patients (PD) and healthy controls (HC), that were assessed by the 10 summarized Parkinson’s disease studies. > symbolizes a higher abundance in HC when compared to PD; < symbolizes a lower abundance in HC when compared to PD; empty cells symbolize, that no significant change in PD compared to HC was found with the chosen methodological approach. The methods used by the studies are Yakult Intestinal Flora-SCAN (YF), 96-well block of the ABI PRISM 7900HT Sequence Detection System (PR), Illumina Miseq sequencing (Ill Miseq), Illumina Hiseq sequencing (Ill Hiseq), Roche 454 GS FLX Titanium sequencing (Ro).

## Appendix A

**Table A1:** Changes in relative abundances in neurodegenerative diseases.

Phylum and Family	Genus (Species, OTU)	AD	PD	MSA	NMO	MS	ALS	Reference
Unclassified bacteria								PD: [39]
Firmicutes								
Unspecified								AD: [56], PD: [38], MSA: [47], ALS: [45]
unclassified								PD: [39], NMO: [58]
								NMO: [58]
91otu15265	o1 = one OTU							NMO, o1: [58]
Lactobacillaceae	Unspec.							PD: [41], [35], [36]
								PD: [43]
	*Lactobacillus* (m *= mucosae*, g *= gasseri*, c *= casei*, f *= fermentum*, re *= reuteri*, ru *= ruminis)*, r *= rogosae*		+			+		PD:, [35] +m: [34], +g, c, f, re, ru: [42], MS: [66], r: [57]
Enterococcaceae	Unspec.							PD: [36], [37]
								PD: [43]
	Enterococcus							PD: [37]
Ruminococcaceae	Unspecified O1 = one OTU		+					AD: [55] PD:, o1: [35], [41] MSA: [47], MS: [75] ALS: [46]
	unclassified						^	PD: [35], NMO: [58], ALS: [71] detected differences in certain unculturable Ruminococcaceae in ALS patients but did not describe the scale and direction.
	*Ruminococcus* b *= bromii* c *= callidus* o38 = 38 OTUs		+					PD, b: [34], NMO: [58], MS, o38: [65]
			+					PD: [37], c: [34] ALS: [46]
	*Papillibacter cinnamivorans*							PD: [34]
	*Faecalibacterium* p *= prausnitzi* o2 = two OTU o57 = 57 OTU		+			+		PD: +o2: [35] [34], [37], p [43] MS, +p: [57], o57: [65]. ALS: [45]
Lachnospiraceae	Unspec.							PD: [35], MSA: [47] MS: [67], ALS: [45],
	Unclassified o3= three OTU		+		+			PD, o3: [35], NMO, o1: [58]
	*Lachnospira,*							ALS: [45]
	*Roseburia* o2 = two OTU		+					PD: [38], +o2: [35], MS: [57]
	*Coprococcus*, e *= eutactus* o1 = one OTU		+					PD: [38], e: [34], o1: [35], NMO: [58]
	*Blautia*, g *= glucerasea*, p *= producta* o1 = one OTU		+		+			AD: [56], PD: [38], [37] +o1: [35], g: [34], MSA: [47] NMO, p,o1: [58], MS: [66]
	*Dorea*, l *= longicatea*		+					PD: +l [34], MSA: [47], MS: [66] ALS: [45]
	*Anaerostipes*, h *= hadrus*					+		MS, +h: [57], ALS: [45]
Catabacteriaceae	*Catabacter*, h *= honkogenesis*		+					PD, +h: [34]
Clostridiaceae	Unspec.							AD: [56] MSA: [47]
	*SMB53*							AD: [56]
	*Anaerotruncus*							PD: [40]
	*Clostridium*, p *= perfringens* s *= saccharolyticum*		+		+			AD: [56], PD, s: [42], [39], NMO, p: [58], MS: [57]
Eubacteriaceae	*Eubacterium*, r *= rectale*, b *= biforme*		+^			+		PD, +b: [39] ^ symbolizes, that Eubacterium is classified as Erysipelotrichoceae in the original publication, MS, r: [57],
	[Candidatus Stoquefichus massiliensis]							PD: [34]
Erysipelotrichaceae	*Unspec.*							PD: [37]
	*cc115*							AD: [56]
Christensenellaceae	unspecified							PD: [35]
	unclassified							PD: [35]
	*Christensenella*, m *= minuta*		+					PD, +m: [34], MS: [67]
Gemellaceae	*Unspec*							AD: [56]
	*Gemella*							AD: [56]
Mogibacteriaceae	*Unspec.*							AD: [56]
Oscillospiraceae	*Oscillospira*							PD: [34], [38],
	*Oscillobacter*							ALS: [45]
Streptococcaceae	*Unspec.*							PD: [37]
	*Streptococcus* t/s *= thermophilus/salivarius*					+		PD: [37], MS, t/s: [57]
Peptococcus	*Desulfotomaculum sp. CYP1*							MS: [57]
Peptostreptococcaceae	*Unspec.*							AD: [56]
Acidaminococcaceae	*Phascolarctobacterium*							AD: [56]
	*Acidaminococcus*							PD: [37]
Veillonellaceae	*Unspec.*							PD: [37]
	*Megamonas*, f *= funiformis YIT11815*							PD: [37],
						+		MS, f: [57]
	*Megasphera*							PD: [37]
	*Dialister*							AD: [56]
Tissierellaceae	*Tissierella*							PD: [35]
Turicibacteraceae	*unspec.*							AD: [56]
	*Turicibacter*							AD: [56]
Tenericutes								
Unclassified								NMO: [58]
Acholeplasmataceae	*Acholeplasma*							NMO: [58]
	[Candidatus Phytoplasma]							NMO: [58]
Melainabacteria								
OTU_171 (98.9% identity to MelB1,57)								PD, o1: [40]
Actinobacteria								
Unspec.								AD: [56], PD: [37], MS: [67]
Bifidobacteriaceae	Unspec.							AD: [56], PD: [35]
	*Bifidobacterium*							AD: [56], PD: [34], [35], [43], MS: [67]
Coriobacteraceae	*Unspec.*							PD: [37]
	*Adlercreutzia*							AD: [56], MS: [66]
	*Collinsella*							MS: [66], [64]
	*Slackia*							MS: [64]
	*Eggerthella lenta*							MS: [57]
Corynebacteriaceae	*Corynebacterium*							NMO: [58]
Fibrobacters								
unclassified								NMO: [58]
Gemmatimonades								
91otu1 0683	o1 = one OTU							NMO, o1: [58]
Bacteroidetes								
Unspec.								AD: [56] PD: [38] MSA: [47], ALS: [45]
								PD: [43], [37]
unclassified								NMO: [58]
Bacteroidaceae	Unspec. o37= 37 OTUs					+		AD: [56] MS, o37: [65]
	*Bacteroides* f *= fragilis*, m *= massiliensis*, cc *= coprocola*, cp *= coprophilus*, d *= dorei*, p *= phlebeus*, s *= stercoris*			+				AD: [56], PD: [38], MSA, f: [70], NMO: [58], MS: [67]
			+					PD, +m, c, d, p: [34], f: [42], MS, s, cc, cp: [57], ALS: [45]
Flavobacteriaceae	*Flavobacterium*							NMO: [58]
Prevotellaceae	Unspec							PD: [41]
	*Prevotella*, co *= copri*, cl *= clara*, M *= melaninogenica* o1 = one OUT, o2 = two OTU		+	+	+	+		PD,+ co: [39], [34] MSA, cl: [70] NMO, co, o2: [58] MS, +co: [57], [64], ALS: [45]
			+		+			PD, o1: [35] NMO, m: [58]
Porphyromonadaceae	Unspec.							MSA: [47],
	Unclass.							NMO: [58]
	*Parabacteroides*							MS: [66]
								PD: [35], MS: [67]
	*Barnesiella*							PD: [36]
	*Butyricimonas*							MS: [64]
Rikenellaceae	Unspec							AD: [56], MSA: [47]
	*Alistipes*, s *= shahii*		+					AD: [56], PD, s: [39]
Sphingobacteriaceae	*Pedobacter*							MS: [66]
Elusimicrobia								
91otu12128	o1 = one OTU							NMO, o1: [58]
Plantomycetes								
unclassified								NMO: [58]
Verrucomicrobia								
Unspec								PD: [40], MS: [64]
Verrucomicrobiaceae	Unspec.							PD: [35] [41] [38] [40]
Akkermansiaceae	*Akkermansia*, m *= muciplila*		+					PD: [35], [38], +m: [39] [40], MS: [64], [67]
[Chthoniobacteraceae]	DA101 = one OTU							NMO, o1: [58]
Acidobacteria								
91oto412	One OTU							NMO, o1: [58]
Spirochaetes								
Spirochaetaceae	*Treponema socranskii*							NMO: [58]
Proteobacteria								
unclassified								PD: [38], [37]
Alpha-Proteobacteria								
Unclassified								NMO: [58]
Bradyrhizobiaceae	*unspec*							PD: [41]
Brucellaceae	*Mycoplana*							MS: [66]
OTU_469, o1=one OTU								PD, o1: [40]
Beta-Proteobacteria								
Sutterellaceae	*Sutterella*, w *= wadsworthensis*					+		MS, w: [57]
Burkholderiacea	*Ralstonia*							MSA: [47]
Oxalobacteraceae	Unspec.							MSA: [47]
Gamma-Proteobacteria								
Unclassified								NMO: [58]
Chromatiaceae	o1 = one OTU.							NMO, o1: [58]
Coxiellaceae	o1 = one OTU							NMO, o1: [58]
Enterobacteriaceae	Unspec.							PD: [43], [37]
	Unclassified							NMO: [58]
	*Escherichia*							ALS: [45]
	*Escherichia/Shigella*							PD: [37]
	*Proteus*							PD: [37]
Moraxellaceae	Unpec.							PD: [37]
	*Acinetobacter*							PD: [37]
Pasteurellaceae	Unspec.							PD: [35]
Pseudomonadaceae	Pseudomonas							MS: [66]
Delta-Proteobacteria								
Desulfovibrionaceae	*Bilophila*							AD: [56]
	*Desulfovibrio*							MS: [67]
KSB3								
unclassified								NMO: [58]
91otu6419	97otu28635 = one OTU							NMO, o1: [58]

AD: Alzheimer’s disease; PD: Parkinson’s disease; MSA: multiple sclerosis; NMO: neuromyelitis optica; MS: multiple sclerosis; ALS: amyotrophic lateral sclerosis; o1,2,3, symbolise that the change is concerning one, two, three or more operational taxonomic units (OTUs); letter = abbreviation for the species in which the change was observed; Small letters followed by a + indicate, that the change was observed for genus and species; Changes in relative abundance compared to the control, are colour coded. Green: Higher abundance in disease condition. Orange: Lower abundance in disease condition. Uncoloured: No study detected a significant change in abundance; - means no significant change in PD compared to HC was found with the chosen methodological approach. There are three intensity nuances for each colour to show how many of the included study found the same difference and direction. Light: one study; Middle: two studies; Dark: three or more studies. Coloured fields containing a + indicates, that detailed information concerning species type is found in the other columns.
